# Isolation of a diazinon-degrading strain *Sphingobium* sp. DI-6 and its novel biodegradation pathway

**DOI:** 10.3389/fmicb.2022.929147

**Published:** 2022-08-23

**Authors:** Guangli Wang, Xiang Li, Jiaxin Zheng, Xuedong Li, Lingling Bai, Wenlong Yue, Li Jiang

**Affiliations:** ^1^Anhui Province Key Laboratory of Pollutant Sensitive Materials and Environmental Remediation, School of Life Sciences, Huaibei Normal University, Huaibei, China; ^2^Laboratory of Applied Microbiology and Biotechnology, School of Pharmaceutical Engineering & Life Science, Changzhou University, Changzhou, China; ^3^State Key Laboratory of Desert and Oasis Ecology, Xinjiang Institute of Ecology and Geography, Chinese Academy of Sciences, Ürümqi, China

**Keywords:** *Sphingobium* sp. DI-6, diazinon-biodegradation metabolites, novel metabolic pathway, 16S rRNA, environmental remediation

## Abstract

Diazinon is one of the most widely used organophosphate insecticides, one that is frequently detected in the environment. In this study, a diazinon-degrading bacterium, DI-6, previously isolated from diazinon-contaminated soil in China has been subsequently identified as *Sphingobium* sp. on the basis of its physiological and biochemical characteristics, as well as by virtue of a comparative analysis of 16S rRNA gene sequences. This strain is capable of using diazinon as its sole carbon source for growth and was able to degrade 91.8% of 100 mg L^–1^ diazinon over a 60-h interval. During the degradation of diazinon, the following seven metabolites were captured and identified by gas chromatography/mass spectrometry (GC–MS) analysis: diazoxon, diazinon aldehyde, isopropenyl derivative of diazinon, hydroxyethyl derivative of diazinon, diazinon methyl ketone, *O*-[2-(1-hydroxyethyl)-6-methylpyrimidin-4-yl] *O*-methyl *O*-hydrogen phosphorothioate, and *O*-(6-methyl pyrimidin-4-yl) *O,O*-dihydrogen phosphorothioate. Based on these metabolites, a novel microbial biodegradation pathway of diazinon by *Sphingobium* sp. DI-6 is proposed. This research provides potentially useful information for the application of the DI-6 strain in bioremediation of diazinon-contaminated environments.

## Introduction

Diazinon [*O,O*-diethyl *O*-(2-isopropyl-6-methyl-4-pyrimidinyl) phosphorothioate] is an organophosphate insecticide in widespread use, with both agricultural and non-agricultural applications; worldwide, two to four million pounds are deposited into the environment, annually (Mahiudddin et al., 2014). It is used as an insecticide and acaricide to control a variety of sucking and chewing animal parasites, for example, as a sheep dip to control ectoparasites such as sheep scab and blowfly strike (Kim et al., 2006). Additionally, it is utilized against a range of crop parasites and also for the control of agricultural soil-dwelling insects. Its widespread use against insects and arachnids, especially in animal parasitism, has led to its identification as a potential chemical mutagen that acts as a contact stomach and respiratory poison (Bolognesi and Morasso, 2000). Additional studies have demonstrated diazinon’s immunotoxicity (Neishabouri et al., 2004), cytotoxicity (Giordano et al., 2007), and genotoxicity (Çakir and Sarikaya, 2005). Given these toxic properties and the potential toxicity of diazinon pose a threat to human health, it is important to trace the entire process of diazinon-degradation and dissipation of diazinon residues.

The removal of diazinon from contaminated soil or other contaminated substrates can usually be achieved through several processes, either alone or in combination, including chemical hydrolysis, volatilization, photolysis, or hydrolysis, and also through microbial degradation (Mahiudddin et al., 2014). However, microbial degradation is considered to be the most effective, eco-friendly, and technically challenging approach for the decontamination of soil, sediment, bodies of water, and other substrates. At present, several isolated bacterial species that have great potential to accomplish diazinon degradation and its use as the sole carbon source belong to different taxonomic groups, including *Flavobacterium* sp. (Ghassempour et al., 2002), *Serratia* sp. (Cycoń et al., 2009; Abo-Amer, 2011), *Burkholderia* sp. (Mahiudddin et al., 2014), and *Pseudomonas* sp. (Cycoń et al., 2009; Mahiudddin et al., 2014). However, details of the pathway for diazinon degradation by these bacteria have not yet been completely established in the research literature. According to most reports to date, diazinon is usually hydrolyzed to diazoxon, a toxic metabolite, and 2-isopropyl-6-methyl-4-hydroxypyimidine (IMHP or oxypyrimidine), a persistent but less-toxic product (Karpouzas and Singh, 2006; Basfar et al., 2007).

This work describes the isolation and characterization of a diazinon-degrading strain of *Sphingobium* sp., referred to as DI-6, which can utilize diazinon as its sole carbon source for growth. Factors influencing the specifics of DI-6’s degradation in liquid medium were examined, and diverse novel diazinon biodegradation pathways, including several newly characterized intermediates, were proposed for the first time. It is hoped that this work will aid the development of a set of guidelines for the risk assessment of pesticides in the environment and also for contaminated soil.

## Materials and methods

### Chemicals and media

Diazinon (98% purity) was purchased from Aladdin Chemical Group Co., Ltd., Shanghai, China. All other chemicals used in this study were of analytical grade and were purchased from Sinopharm Chemical Reagent Co., Ltd. (Shanghai China).

Mineral salts medium (MSM) consisted of the following components: 1.0 g of NH_4_NO_3_, 1.0 g of NaCl, 1.5 g of K_2_HPO_4_, 0.5 g of KH_2_PO_4_, and 0.2 g of Mg_2_SO_4_.7H_2_O per liter of deionized water. The final *pH* value was adjusted to 7.2. Luria–Bertani (LB) medium contained 10 g of peptone, 10 g of NaCl, and 5 g of yeast extract, with distilled water added to adjust the final volume to 1 L. Solid medium plates were prepared by adding 1.5% wt vol^–1^ agar to the liquid medium. After autoclaving (121°C, 30 min) and cooling, the medium was supplemented with a diazinon solution, as described below.

### Enrichment and isolation of diazinon-degrading bacteria

A soil sample was collected from an agricultural field in Nanjing, Jiangsu Province, China. The field has been managed with pesticides for many years, and it has been found to support microbial degraders that have some specificity for diazinon. Diazinon was dissolved in acetone, sterilized by filtration, and subsequently added to the sample at different concentrations in the MSM medium. To isolate diazinon-degrading organisms, approximately 10 g of the soil sample was added to a 250-ml Erlenmeyer flask containing 100 ml of MSM medium with the addition of diazinon (50 mg L^–1^) as the sole carbon source; this mixture was incubated in a rotary shaker at 160 rpm for 7 days. Every 7 days, 5 ml of the enriched samples were transferred to 100 ml fresh MSM medium with a stepwise increase in diazinon concentration to 200 mg L^–1^. The enrichment culture that exhibited the ability to degrade diazinon was serially diluted and spread onto MSM agar plates. After incubation at 30°C for 10 days, colonies were picked and further purified by repeated streaking, then tested for diazinon-degrading capabilities through a combination of UV–vis spectrophotometry (SHIMADZU Corp., Kyoto, Japan) and high-performance liquid chromatography (HPLC) analysis. One strain, which exhibited the highest diazinon-degrading capability, was designated DI-6 and was selected and reserved for further investigation.

### Identification and characterization

The DI-6 isolate was characterized on the basis of its morphological, physiological, and biochemical properties according to *Bergey’s Manual of Determinative Bacteriology* (Holt et al., 1994). In addition, 16S rRNA gene-based molecular phylogenetic analyses were carried out, toward the identification of the bacterial strain. Genomic DNA was extracted (Miller et al., 1988) and the 16S rRNA gene was amplified by polymerase chain reaction (PCR) using the universal eubacterial primers: 27F (5′-AGAGTTTGATCCTGGCTCAG-3′), forward and 1492R (5′-TACGGTTACCTTGTTACGACTT-3′), reverse. The 50-μl reaction mixture was composed of the following components: 1 μl total DNA as template; dNTP (2.5 mM) 1 μl, primer (1 mM) each nucleotide, 1 μl; 10 × Buffer, 5 μl; MgCl_2_ (5 mM), 3 μl; Taq Enzymes (5 U μl^–1^), 0.5 μl; and ultra-pure water, 37.5 μl. Amplification was conducted using a PCR Master Mix Kit (Promega, Madison, WI, United States) according to the manufacturer’s instructions, and a PTC-118 Thermal Cycler (BIO-RAD, Irvine, CA, United States), under the following conditions: (1) an initial denaturation step of 95°C for 3 min; (2) 30 cycles of denaturation, annealing, and extension (95°C for 1 min, followed by 52°C for 1 min, with an extension step at 72°C for 1.5 min); and (3) a final extension at 72°C for 10 min (Sambrook and Russell, 2001). PCR products were purified using the QIAquick PCR Purification Kit (Qiagen, Valencia, CA, United States) before the sequencing of the amplicons. The nucleotide sequence coding for the 16S rRNA gene of DI-6 was sequenced (Sangon, Shanghai, China). Pairwise sequence similarity was calculated by using a global alignment algorithm, implemented at the EzTaxon server (Chun et al., 2007). Phylogenesis was analyzed with MEGA version 6.0 software and the distance was calculated using the Kimura 2 parameter distance model (Thompson et al., 1997). A phylogenetic tree was built using the neighbor-joining method. Each dataset was bootstrapped 1,000 times.

### Growth and biodegradation experiments

#### Sample preparation

In preparation for further analysis of other inocula, isolates that had previously been selected were inoculated onto LB agar plates supplemented with 300 mg L^–1^ diazinon and incubated at 30°C for 5 days. A pure culture of each isolate, obtained from individual colonies, was inoculated into a 250-ml Erlenmeyer flask containing 100 ml of LB liquid medium and incubated on a rotary shaker at 160 rpm for 3 days. The cell density (OD_600 nm_) was measured using a UV–vis spectrophotometer. All experiments were carried out in triplicate. During the exponential phase, the bacteria were harvested by centrifugation (5 min, 3,000 rpm). The pellet was gently rinsed with sterile normal saline and was suspended in normal saline, to prepare a bacterial suspension.

#### Biodegradation of diazinon by strain DI-6 in liquid medium

After the cell density had been adjusted to approximately 1.0 × 10^8^ CFU ml^–1^ (Inorganic salt medium), the aliquots (5%, v/v) were inoculated into 500-ml conical flasks containing 100 ml of MSM supplemented with 300 mg L^–1^ diazinon. Samples of MSM supplemented with diazinon but free of bacterial inoculation were retained as controls. Conical flasks were incubated at 30°C on a rotary shaker at 160 rpm, in the dark to avoid photodegradation of diazinon. Samples of liquid medium were aseptically withdrawn at regular intervals to assess both bacterial growth (OD_600 nm_) and diazinon degradation; diazinon degradation efficiency was estimated by the loss of diazinon from the culture. DI-6 cell growth was also investigated to determine whether the strain could utilize diazinon as the sole carbon source. Cell density was monitored by measuring the absorbance at 600 nm using a SHIMADZU UV–vis recording spectrophotometer.

#### Effects of including both diazinon and glucose in growth media

To investigate the effect of diazinon concentration on the overall rate and pattern of degradation, diazinon was initially utilized as the sole carbon source in MSM. Conical flasks (500 ml) containing 100 ml of MSM supplemented with concentrations of diazinon, of 100, 200, 300, 400, and 500 mg L^–1^, respectively, were inoculated with 10 ml strain DI-6 and incubated as described above. At designated intervals, samples were withdrawn for the analysis of diazinon degradation.

Glucose was included as the standard carbon source, readily utilized by most soil bacteria (Caulfield et al., 2003). To study the effect of glucose on diazinon biodegradation, the medium was supplemented with 100, 250, 500, and 1,000 mg L^–1^ of glucose, respectively. Conical flasks (500 ml) containing 100 ml of MSM supplemented with different concentrations of glucose were inoculated with 10 ml strain DI-6 and 300 mg L^–1^ diazinon. All treatments were replicated in triplicate, and the control without glucose was treated under the same conditions as the experimental cultures.

In these two experiments, an initial bacterial inoculum with a cell wet weight of 3 × 10^2^ to 5 × 10^2^mg L^–1^ was applied in each flask. Unless otherwise noted, cultures were incubated on a rotary shaker at 160 rpm for 7 days at 30°C.

### Chemical analysis

Extraction of diazinon from the bacterial cultures was performed using an equal volume of dichloromethane. This extract was dried over anhydrous Na_2_SO_4_ and evaporated using a vacuum rotary evaporator at standard room temperature. The residue was then dissolved in an equal volume of methanol. Samples were analyzed by high-performance liquid chromatography (HPLC), utilizing a 600 controller, Rheodyne 7725i manual injector, and 2487 Dual k Absorbance Detector (Waters Co., Milford, MA, United States). Kromasil 100-5 C18 stationary phase was used in the separation column (4.6-mm internal diameter and 25 cm length). The extract was then dried as described above and re-dissolved in an equal volume of the following mobile phase: acetonitrile water (70:30, v/v), with the flow rate 1.0 ml min^–1^, and injection volume of 20 μl. Diazinon was detected at 254 nm.

Statistical analysis data were expressed as mean ± S.D. of three replicates. Statistical analyses were performed with R, version 3.5.3.

### Identification of the metabolites resulting from diazinon degradation

To identify the metabolites deriving from diazinon biodegradation, strain DI-6 was inoculated into 100 ml MSM medium with 300 mg L^–1^ diazinon at 30°C. Negative control included the same components, except that the DI-6 cells had been heat-killed before inoculation. Triplicate samples were collected at 16, 48, and 72 h, respectively, and triplicate samples from each time point were pooled and extracted with an equal volume of dichloromethane. All organic phase extracts were then evaporated as described above and subsequently re-dissolved in a total of 2 ml of methanol. The combined extracts were then separated and identified by GC–MS (Thermo-Finnigan, Ringoes, NJ, United States).

The GC–MS analyses were performed in electron ionization (EI) mode (70 eV), utilizing a Finnigan GC, equipped with an MS detector. A Finnigan capillary column (30-m length × 250-μm inside diameter × 0.25 μm film thickness) was used with the following temperature program: 110°C for 1 min; increased to 200°C for 3 min at 20°C min^–1;^ then increased to 250°C for 2 min at 5°C min^–1^; and finally, increased to 270°C for 1 min at 10°C min^–1^. The helium carrier gas was set at constant pressure mode with a pressure of 13.55 psi and a constant flow of 3.0 ml min^–1^. The sample solution (1 μl) was analyzed in split mode (15:1) at an injection temperature of 230°C and an EI source temperature of 230°C and scanned in the mass range from 30 to 650 *m/z*.

## Results and discussion

### Isolation and identification of diazinon-degrading strain DI-6

Through initial utilization of the enrichment culture technique, several bacterial strains were isolated from the long-term diazinon-contaminated soil samples. All isolated strains were tested for their degrading capabilities. Strain DI-6 emerged because of its ability to nearly completely degrade 91.8 mg L^–1^ diazinon in MSM liquid medium over a 60-h period. It exhibited growth at temperatures ranging from 20 to 37°C, and at *pH* values from 6.0 to 9.0. On LB agar, DI-6 colonies appeared focally dense, semi-transparent, and pale yellow, emitting water-soluble pigment after 3 days of incubation at 30°C. Results from biochemical tests identified DI-6 as Gram-negative, non-motile, non-sporulating, and with rod-shaped morphology. This bacillus tests positive for β-galactosidase and the metabolic assimilation of D-glucose, L-arabinose, and maltose; it can hydrolyze aesculin, but tests negative for catalase, urease, and arginine dihydrolase.

**FIGURE 1:**
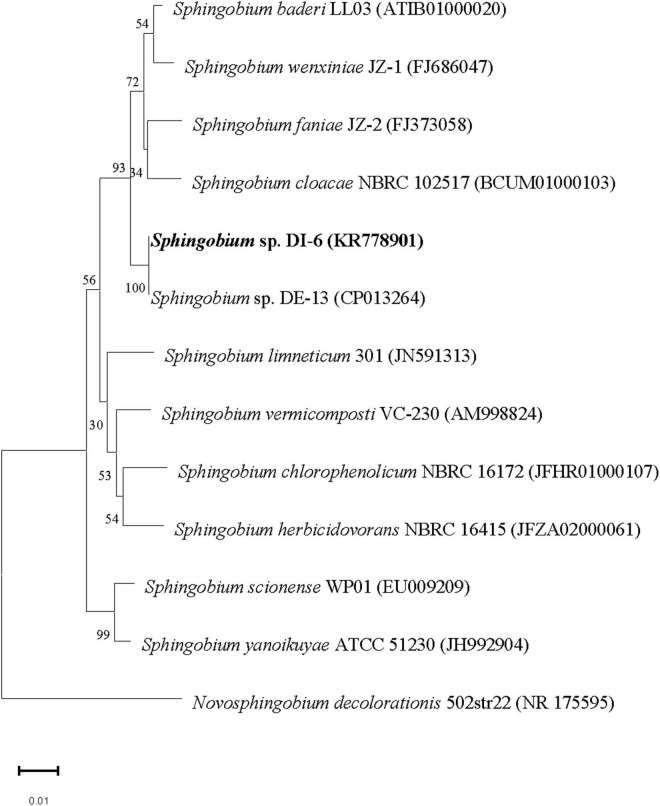
Phylogenetic analysis of strain DI-6 and related species by the neighbor-joining approach. Bootstrap values obtained with 1,000 resamplings are indicated as percentages at all branches. A sequence of *Novosphingobium decolorationis* was used as the out-group. The scale bars represent 0.01 substitutions per nucleotide position. The GenBank accession number for each strain is shown in parentheses after the species name.

The nucleotide sequence encoding DI-6 16S rRNA was deposited in the GenBank database under accession number KR778901. Multiple alignments revealed that DI-6’s 16S rRNA gene sequence was closely related to that of *Sphingobium baderi* LL03*^T^* (98.9% similarity; Kaur et al., 2013), *Sphingobium wenxiniae* JZ-1*^T^* (98.5% similarity; Wang et al., 2011), and *Sphingobium faniae* JZ-2*^T^* (98.4% similarity; Guo et al., 2010). From molecular phylogenetic analyses, it is clear that the strain DI-6 belonged to the genus *Sphingobium*. Currently, overall genome relatedness values and whole genome–derived ANI values are considered index for predicting the species status of a bacterial strain (Wayne et al., 1987; Kim et al., 2014). Moreover, the extent of sequence identity of the strain DI-6 with closest phylogenetic relatives was above the threshold values recommended for delineation of species in prokaryotes (Stackebrandt and Goebel, 1994; Kim et al., 2014). Above this threshold value, rRNA gene sequence identity loses its resolving capacity and thus the value of overall genome relatedness has to be determined to ascertain the species status. In the presence of a high sequence identity value (with closest phylogenetic relative that is above the threshold value) and the absence of overall genome relatedness values, ANI values, and detailed characterization (as per the polyphasic taxonomic approach), its species status could not be determined. Therefore, the strain DI-6 was identified as *Sphingobium* sp. On the basis of morphological, physiological, and biochemical properties, and this phylogenetic analysis of the 16S rRNA gene sequence (Figure 1), strain DI-6 was identified as *Sphingobium* sp. Members of this genus are found to be saprophytic soil and water bacteria, and many isolates have been shown to engage in the biodegradation of a variety of toxic organic contaminants such as nonylphenol (Ushiba et al., 2003), phenanthrene (Prakash and Lal, 2006), polycyclic aromatic compounds (Wittich et al., 2007), pentachlorophenol (Zilouei et al., 2008), pyrethroids (Guo et al., 2009), isoproturon (Sun et al., 2009), lindane (Zheng et al., 2011), phenanthrene (Wang et al., 2013), organophosphate and organochlorine pesticides (Cao et al., 2013), and a mixture of polycyclic aromatic hydrocarbons (Fu et al., 2014). *Sphingobium* sp. DI-6 degraded diazinon rapidly, indicating the strain’s significant potential for removing diazinon residues from the environment as well as from agricultural products.

### Degradation of diazinon by strain DI-6 in liquid culture

**FIGURE 2:**
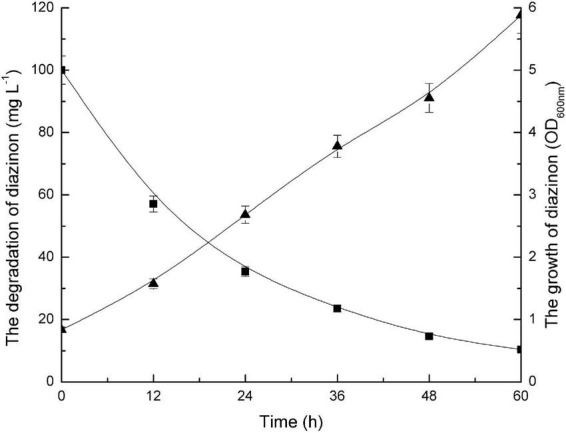
Growth of bacterial isolate (DI-6) and degradation of diazinon. Diazinon concentration and DI-6 cell growth in mineral salts medium (MSM) supplemented with 100 mg L^– 1^ diazinon at 30°C (◼, diazinon concentration; ▲, cell growth). The data are represented as the mean and standard deviation for triplicate incubations.

Figure 2 illustrates both the time course of diazinon degradation and the kinetics of DI-6 cell growth in MSM containing 100 mg L^–1^ diazinon. It was recorded that 100 mg L^–1^ diazinon was degraded to 8.2 mg L^–1^ within 60 h. As measured by OD_600 nm_, DI-6 cell density increased from 0.837 to 5.882 during diazinon degradation, indicating that strain DI-6 was able to utilize diazinon as its sole carbon source for growth. In contrast, in the non-inoculated control, no changes in growth or diazinon concentration were observed during the experimental period.

Additional studies on the degradation characteristics showed that DI-6 could efficiently degrade diazinon at temperatures ranging from 20 to 37°C and *pH* values ranging from 6.0 to 9.0. The optimal conditions for diazinon biodegradation were 30°C and pH 7.0.

### Effect of diazinon concentration on degradation

**FIGURE 3:**
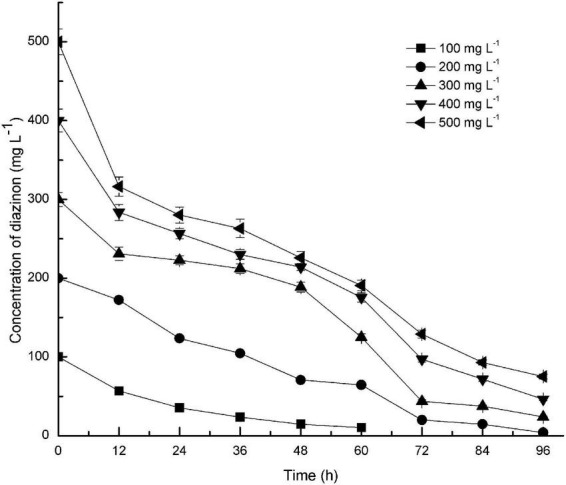
Effect of initial substrate concentrations on the biodegradation of diazinon by DI-6. Error bars, mean ± SD of three replicates. Each symbol represents the substrate concentration of diazinon; Square: 100 mg L^−1^ of diazinon was added; Circle: 200 mg L^−1^ of diazinon was added; Upper triangle: 300 mg L^−1^ of diazinon was added; Lower triangle: 400 mg L^−1^ of diazinon was added; Left triangle: 500 mg L^−1^ of diazinon was added.

Diazinon degradation kinetics by DI-6 were contingent on the initial diazinon concentration. As illustrated by Figure 3, DI-6 degraded and utilized diazinon up to a concentration of 500 mg L^–1^, however, at higher diazinon concentrations, the degradation lagged behind that observed at lower concentrations. At an initial diazinon concentration of 100 mg L^–1^, the degradation rate was such that 100% was degraded by 72 h, and at an initial concentration of 200 mg L^–1^, 90% ± 3.9% (*p* < 0.05 by Student’s *t*-test) was degraded within 72 h, a 10% decrease in degradation rate compared to the initial concentration of 100 mg L^–1^. However, when the concentration was increased above 200 mg L^–1^, only 85.5% ± 2.7% (*p* < 0.05 by Student’s *t*-test), 75.7% ± 3.3% (*p* < 0.05 by Student’s *t*-test), and 74.2% ± 2.8% (*p* < 0.05 by Student’s *t*-test) was degraded within 72 h, for initial concentrations of 300, 400, and 500 mg L^–1^, respectively, which was significantly lower degradation rate than that of the initial diazinon concentration of 100 mg L^–1^, with complete degradation requiring a longer interval. Nonetheless, this is the first report of the isolation of a soil bacterium with the capability of degrading diazinon with such rapid kinetics. Previous results reported that the isolates *Serratia liquefaciens*, *S. marcescens*, *Pseudomonas* sp., and their consortium required 14 days in which to degrade 80–92% of diazinon, when diazinon was added to MSM to yield a concentration of 50 mg L^–1^ (Cycoń et al., 2009). By contrast, at two times the diazinon concentration, DI-6 completely degraded diazinon in 3 days.

### Effect of an extra carbon source on diazinon degradation

**FIGURE 4:**
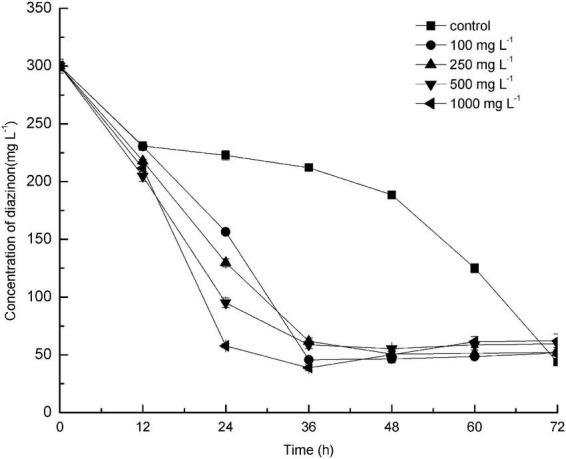
Effect of addition of different concentrations of glucose as co-substrate on the biodegradation of diazinon by DI-6. Error bars, mean ± SD of three replicates. Each symbol represents the concentration of added glucose, Square: control indicates no added glucose; Circle: 100 mg L^−1^ of glucose was added; Upper triangle: 250 mg L^−1^ of glucose was added; Lower triangle: 500 mg L^−1^ of glucose was added; Left triangle: 1000 mg L^−1^ of glucose was added.

The effect of different concentrations of glucose on diazinon biodegradation by DI-6 was also investigated (Figure 4). The diazinon degradation efficiency (the quantity of diazinon degraded per unit time) reached a maximum of 25.7% ± 0.6% degradation per 24 h when 300 mg L^–1^ diazinon was added as sole carbon source (control). In comparison, after adding different concentrations of glucose, degradation efficiency was greatly enhanced within the same 24-h period. However, after an additional 36 h, the diazinon degradation efficiency became almost constant. These results are similar to those of Cai et al.’s (2013), who found that exogenously supplied glucose promoted the degradation of piperazine. Wen et al. (2010) and Wang et al. (2012) demonstrated the opposite effect: the addition of a low concentration of glucose could slightly enhance the biodegradation efficiency, but higher concentrations of added glucose delayed biodegradation, thereby decreasing efficiency (Wen et al., 2010; Wang et al., 2012). Based on these studies, it appears that the addition of certain amounts of an extra carbon source can sometimes enhance biodegradation of toxic compounds, with this effect dependent on both the specific compound being degraded and the bacterial species utilized.

### Identification of metabolites during diazinon biodegradation

**FIGURE 5:**
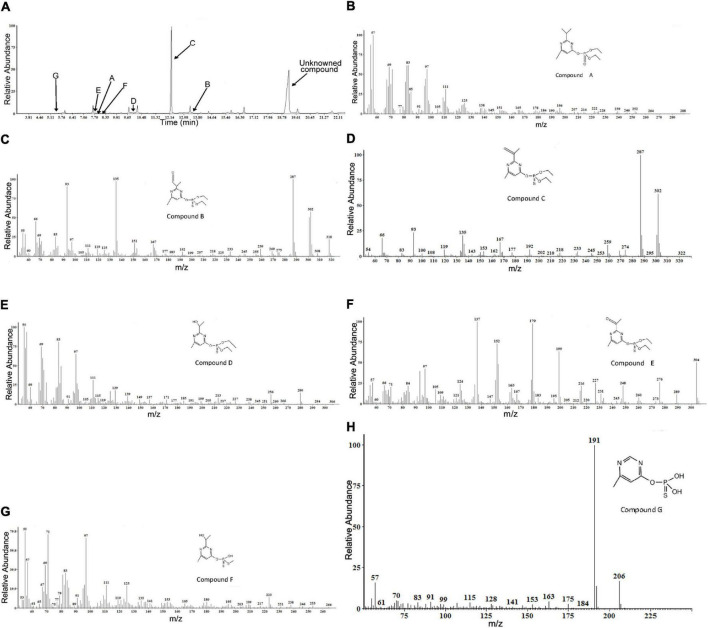
Gas chromatography/mass spectrometry (GC–MS) analysis of the diazinon intermediates transformed by *Sphingobium* sp. DI-6: **(A)** Gas chromatography profiles for main intermediates **(A–G)**. The retention times of the compounds were 7.901, 13.489, 12.246, 9.961, 7.849, 8.301, and 5.468, respectively. **(B–H)** The characteristic ions of compounds **(A–G)** in GC–MS. They were identified as diazoxon, diazinon aldehyde, isopropenyl derivative of diazinon, hydroxyethyl derivative of diazinon, diazinon methyl ketone, *O*-[2-(1-hydroxyethyl)-6-methyl pyrimidin-4-yl] *O*-methyl *O*-hydrogen phosphorothioate, and *O*-(6-methyl pyrimidin-4-yl) *O*,*O*-dihydrogen phosphorothioate, respectively.

Gas chromatography/mass spectrometry analysis of the combined extracts as described above indicated that, during diazinon degradation, many different peaks are recorded, indicating a diversity of diazinon metabolites. In a standard MS analysis (Figure 5A), prominent protonated molecular ions occur at *m/z* = 288 M^+^, *m/z* = 318 M^+^, *m/z* = 302 M^+^, *m/z* = 306 M^+^, *m/z* = 304 M^+^, *m/z* = 264 M^+^, *m/z* = 206 M^+^, and compounds corresponding to the protonated molecular ions are designated as compounds A, B, C, D, E, F, and G. Their retention times were 7.901, 13.489, 12.246, 9.961, 7.849, 8.301, and 5.468 min, respectively.

In addition, another metabolite was found—a compound with a retention time of 19.146 min—that did not match any compound in the National Institute of Standards and Technology (NIST) library, and it was, therefore, designated as an unknown compound. The seven degradation products (A-G) identified for diazinon degradation in liquid culture and their fragment ions and retention times are summarized in Table 1.

**TABLE 1:** Diazinon metabolites identified by gas chromatography/mass spectrometry (GC–MS).

Compound	Chemical name	RT (min)	Characteristic ions in GC–MS (*m*/*z*)
A	diazoxon	7.901	111,125,138,151,264,288
B	diazinon aldehyde	13.489	135,151,245,259,287,302,318
C	isopropenyl derivative of diazinon	12.246	153,245,259,274,287,302
D	hydroxyethyl derivative of diazinon	9.961	139,185,252,280,294,306
E	diazinon methyl ketone	7.849	124,137,152,248,276,304
F	*O*-[2-(1-hydroxyethyl)-6-methyl pyrimidin-4-yl] *O*-methyl *O*-hydrogen Phosphorothioate	8.301	119,135,153,246,264
G	*O*-(6-methyl pyrimidin-4-yl) *O*,*O*-dihydrogen phosphorothioate	5.468	175,191,206

Molecular ion (M^+^) peak of compound A was found to be diazoxon (Figure 5B). The molecular ion (M^+^) peak of compound A (RT = 7.901 min) was 288 *m/z* with characteristic ions at 264 *m/z*—(M^+^-CH_2_CH_3_), 151 *m/z*—[M^+^-O-PO-2(OCH_2_CH_3_)], 138 *m/z*—[M^+^-O-PO-2(OCH_2_CH_3_)-CH_3_], 125 *m/z*—[M^+^-O-PO-2(OCH_2_CH_3_)-2(CH_3_)], and 111 *m/z*—[M^+^-O-PO-2(OCH_2_CH_3_)-CH(CH_3_)_2_]. Additionally, the fragment ions at 264 *m/z* were produced by the loss of an ethyl group from the molecular ion. The fragment ion at 151 *m/z* corresponded to the pyrimidine moiety of diazoxon, and the ions at 138 and 125 *m/z* were related to the loss of a methyl group from the fragment ion at 151 *m/z*. The ion at 111 *m/z* was produced through the removal of a propyl group from the fragment ion at 151 *m/z*.

The (M^+^) peak of compound B (RT = 13.489 min) was 318 *m/z*, with characteristic ions at 302 *m/z*—(M^+^-H), 287 *m/z*—(M^+^-H-CH_3_), 259 *m/z*—(M^+^-H-CH_2_CH_3_), 245 *m/z*—[M^+^-H-CH_3_-(CH_2_CH_3_)], 151 *m/z*—[M^+^-O-PO-2(OCH_2_CH_3_)], and 135 *m/z*—[M^+^-CH_3_-O-PO-2 (OCH_2_CH_3_)] (Figure 5C). According to these results, compound B was identified as diazinon aldehyde. Through oxidation, the fragment ion at 302 *m/z* was produced by replacing a P=S bond with the P=O bond of compound B. The fragment ions at 287 and 259 *m/z* were related to the loss of a methyl group or ethyl group, respectively, from the fragment ion at 302 *m/z*. The fragment ion at 245 *m/z* was produced by the loss of a methyl group from the fragment ion at 259 *m/z*. Similarly, the fragment ion at 151 *m/z* corresponded to the pyrimidine moiety of diazinon aldehyde, and the ion at 111 *m/z* was produced through the removal of a methyl group from the fragment ion at 151 *m/z*.

Compound C (RT=12.246 min) was identified as an isopropenyl derivative of diazinon [*O,O*-diethyl *O*-(2-isopropenyl-6-methylpyrimidin-4-yl) thiophosphate]. The (M^+^) peak of compound D was 302 *m/z*, with characteristic ions at 287 *m/z*—(M^+^-CH_3_), 274 *m/z*—(M^+^-CH_2_ CH_3_), 259 *m/z*—[M^+^-CH_3_-(CH_2_CH_3_)], 245 *m/z*—[M^+^-2(CH_2_CH_3_)], and 153 *m/z*—[M^+^-O-PO- 2(OCH_2_ CH_3_)] (Figure 5D). The fragment ions at 287 and 274 *m/z* were formed by the loss of one methyl or ethyl group from the molecular ion, respectively. The fragment ion at 259 *m/z* was produced by removal of one methyl and one ethyl group from the molecular ion, respectively; additionally, the fragment ion at 245 *m/z* was formed through the loss of an ethyl group, two times. Additionally, the fragment ion at 153 *m/z* corresponded to the pyrimidine moiety of the isopropenyl derivative of diazinon.

Compound D (RT=9.961 min) exhibited a prominent protonated molecular ion at *m/z* = 306 (M^+^) in standard MS, with characteristic fragment ion at *m/z* = 294—(M^+^-OH), *m/z* = 280—(M^+^-CH_2_CH_3_),252[(M^+^-2(CH_2_CH_3_)], *m/z* = 185—(M^+^-CH_2_CH_3_-H), and *m/z* = 139—[M^+^-CH_3_-O-PS-(OC_2_H_5_)_2_] (Figure 5E). Therefore, it was identified as a hydroxyethyl derivative of diazinon {*O,O*-diethyl *O*-[2-(1-hydroxyethyl)-6-methyl pyrimidin-4-yl] thiophosphate}. The fragment ions at 280 and 252 *m/z* resulted from the loss of an ethyl group from the molecular ion. The formation of ions at 185 *m/z* resulted from the α-cleavage of the molecular ion, with the migration of the ethyl group and the loss of hydrogen. The loss of a hydroxyl group from the molecular ion resulted in the ions at 294 *m/z*. The fragment ion at 137 *m/z* was characteristic of the pyrimidine structure, which was supported by the MS/MS analysis reported by Kouloumbos et al. (2003).

Compound E (RT = 7.849 min) was identified as diazinon methyl ketone [*O,O*-diethyl *O*-(2-acetyl-6-methylpyrimidin-4-yl) thiophosphate], by comparing the mass spectrum with a previously reported mass spectrum of the intermediate during photocatalytic degradation of diazinon (Kouloumbos et al., 2003). Its characteristic fragment ions are found at *m/z* = 276—(M^+^-CH_2_CH_3_), *m/z* = 248—{M^+^-2(CH_2_CH_3_), *m/z* = 152—[M^+^-O-PO-2(OCH_2_CH_3_)], *m/z* = 137—[M^+^-O-PO-2(OCH_2_CH_3_)-CH_3_], and *m/z* = 124—[M^+^-O-PO-2(OCH_2_ CH_3_)-(CH_3_)_2_] (Figure 5F). It exhibited a peak at 304 *m/z* corresponding to the molecular ion, and the characteristic ions at 276 and 248 *m/z* resulting from the loss of one or two ethyl groups from the molecular ion, respectively. The ion at 179 *m/z* was formed through the α-cleavage of the molecular ion, accompanied by ethyl migration; and the ion at 152 *m/z* structurally corresponded to pyrimidine structures. The ions at 137 and 124 *m/z* were related to the loss of a methyl group from the fragment ion at 152 *m/z*.

Compound F (RT = 8.301 min) was identified as *O*-[2-(1-hydroxyethyl)-6-methyl pyrimidin-4-yl] *O*-methyl *O*-hydrogen phosphorothioate. Its characteristic fragment ions are found at *m/z* = 246—(M^+^-CH_3_), *m/z* = 153—[M^+^-O-PO-2(OCH_2_CH_3_)], *m/z* = 135—[M^+^-O-PO-2(OCH_2_CH_3_)-H_2_O], and *m/z* = 119—[M^+^-O-PO-2(OCH_2_ CH_3_)-H_2_O-CH_3_] (Figure 5G). The ion at 264 *m/z* was formed through the loss of a methyl group from the molecular ion. Similarly, the fragment ion at 153 *m/z* corresponded to the pyrimidine moiety of compound F, and the ion at 135 *m/z* was produced by dehydration of the fragment ion at 153 *m/z*. Furthermore, the ion at 119 *m/z* was formed by demethylation of the fragment ion at 135 *m/z*.

Finally, compound G (RT = 5.468 min) was identified as *O*-(6-methyl pyrimidin-4-yl) *O,O*-dihydrogen phosphorothioate. Its characteristic fragment ions are found at *m/z* = 191—(M^+^-CH_3_) and *m/z* = 175—(M^+^-CH_3_-H) (Figure 5H). It exhibited an ion at 191 *m/z*, as the base peak, and a molecular ion at 306 *m/z*. The formation of the ion at 191 *m/z* resulted from demethylation of the molecular ion. The fragment ion at 175 *m/z* was produced by replacing the P=S bond with a P=O bond in the fragment ion at 191 *m/z*.

**FIGURE 6:**
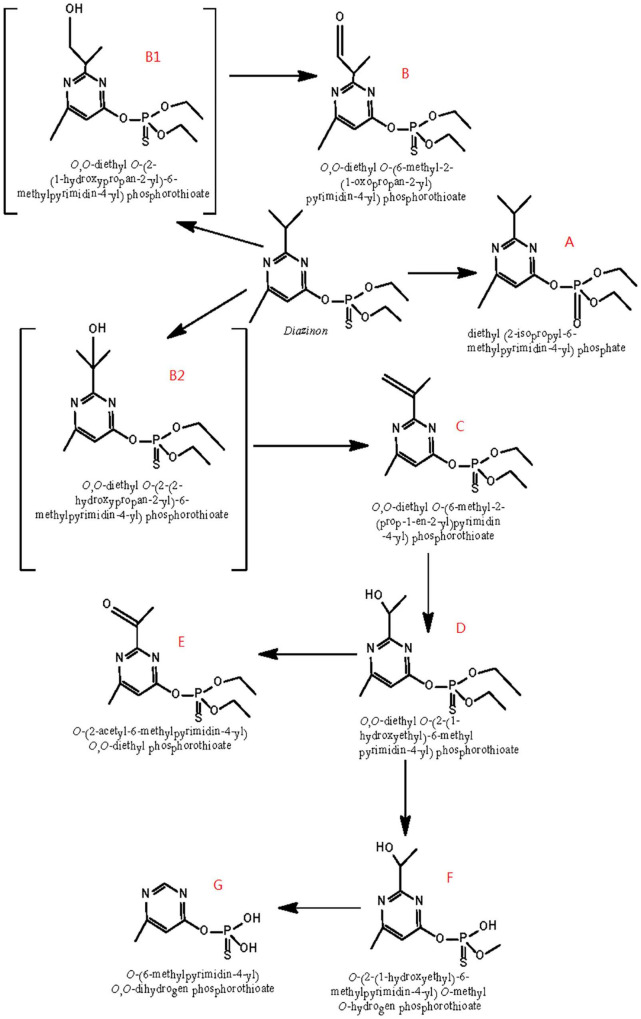
Scheme of the proposed degradation pathway of diazinon in liquid cultures: **(A)** diazoxon; **(B)** diazinon aldehyde; **(C)** isopropenyl derivative of diazinon; **(D)** hydroxyethyl derivative of diazinon; **(E)** diazinon methyl ketone; **(F)** *O*-[2-(1-hydroxye thyl)-6-methyl pyrimidin-4-yl] *O*-methyl *O*-hydrogen phosphorothioate; **(G)** *O*-(6-methyl pyrimidin-4-yl) *O*,*O*-dihydrogen phosphorothioate; B1: 2-hydroxydiazinon {*O*,*O*-diethyl-*O*-[2-(1-hydroxypropan-2-yl)-6-methylpyrimidin-4-yl] phosphorothiotae}; B2: hydroxy diazinon {*O*,*O*-diethyl *O*-[2-(2-hydroxypropan-2-yl)-6-methylpyrimidin-4-yl] phosphorothiotae}.

Based on the identification of degradation products, Figure 6 postulates a degradation pathway for diazinon in liquid cultures. Hydrolysis of the ester moiety, oxidation, hydroxylation, dehydration, demethylation, and decarboxylation are all chemical processes that occur during the degradation of diazinon.

First, the formation of diazoxon (compound A) resulted from the substitution of sulfur by oxygen on the P=S bond through oxidation. In addition, it was inferred that diazinon was transformed to 2-hydroxydiazinon (compound B1) and hydroxy diazinon (compound B2), both of which were not detected, due to hydroxylation at the primary and tertiary carbon atoms of the isopropyl group, respectively. Such a hydroxylation reaction had also been reported during the degradation of contaminants under ultrasonic treatment (Rehorek et al., 2004; Torres et al., 2008). In addition, compound B1 might be transformed into compound B by reduction, removing two hydrogens. Subsequently, *via* dehydration occurring on the 1-hydroxyisopropyl group, hydroxydiazinon (compound B2) would lead to the formation of the isopropenyl derivative of diazinon (compound C). Following this, compound C would be further converted into the hydroxyethyl derivative of diazinon (compound D), probably through oxidation plus decarboxylation, as reported by Miyazaki et al. (1970) in researching the metabolism of diazinon in animals and plants. Furthermore, the hydroxyl group of compound D would be oxidized into a carbonyl group, yielding diazinon methyl ketone (compound E). Compound D could then be transformed into F, possibly through the removal of one ethyl and one methyl group, respectively. Moreover, compound F might become compound G, through the single removal of a hydroxy group and the removal of two methyl groups.

Because IMHP, diethyl phosphate, or diethyl thiophosphate—hydrolysis products of diazinon or diazoxon—were not detected in this study, probably, they could not be easily recovered from extracts of DI-6 diazinon metabolism. Therefore, further research is needed to establish a more thorough identification of these metabolites.

## Conclusion

In this study, strain DI-6 identified as *Sphingobium* sp., isolated from long-term diazinon-contaminated soil, was shown to be capable of diazinon degradation. In MSM medium, DI-6 was able to degrade 91.8% of 100 mg L^–1^ diazinon within 60 h in liquid culture under optimal conditions (pH 7.0, 30°C). The initial concentration of diazinon as well as the presence of a second carbon source (glucose) had a marked effect on the degradation of diazinon. The rate of degradation increased with lower initial diazinon concentrations and with glucose addition, respectively. Seven diazinon-degradation products of strain DI-6’s degradative metabolism have been identified through GC–MS analysis, and four major metabolic pathways including hydrolysis of the ester moiety, oxidation, hydroxylation, dehydration, demethylation, and decarboxylation are proposed, and two novel metabolic products have been identified, not heretofore described. This study offers clarification regarding microorganismal biodegradation of diazinon, including the chemical mechanisms by which biodegradation is accomplished. Further studies will be needed to investigate the potential application of strain DI-6 to the bioremediation of diazinon-contaminated environments.

## Data availability statement

The original contributions presented in this study are included in the article/supplementary material, further inquiries can be directed to the corresponding authors.

## Author contributions

GW conceived and designed the experiments. XL and JZ performed the experiments. GW and LJ wrote the manuscript. GW, WY, XDL, LB, and LJ analyzed the data. All authors reviewed the manuscript and approved the submitted version.
