# Erratum: Isolation of a diazinon-degrading strain *Sphingobium* sp. DI-6 and its novel biodegradation pathway

**DOI:** 10.3389/fmicb.2023.1219721

**Published:** 2023-05-22

**Authors:** Frontiers Production Office

**Affiliations:** Frontiers Media SA, Lausanne, Switzerland

**Keywords:** *Sphingobium* sp. DI-6, diazinon-biodegradation metabolites, novel metabolic pathway, 16S rRNA, environmental remediation

Due to a production error, an author name was incorrectly presented as “Jiang Li.” The correct name is “Li Jiang.”

The publisher apologizes for this mistake. The original article has been updated.

